# Paroxysmal Neuroses

**Published:** 1906-03-10

**Authors:** 


### PAROXYSMAL NEUROSES.

Mr. W. N. Robertson [1] has published a communication dealing with the results obtained by treating a number of paroxysmal neuroses upon the lines suggested by the successful treatment of asthma by intra-nasal methods. In 1871 Voltilini demonstrated the causal relationship between nasal disease and spasmodic asthma, and Dr. H. A. Francis has pursued the practice of intra-nasal treatment with considerable success. In 1903 Francis Hare, of Brisbane, published an account of the mechanism of the paroxysmal neuroses which attributed to them a vaso-motor origin; the author has accepted the explanation, and has attempted the treatment of neuroses analogous to asthma upon similar lines.

Of two hundred and ten cases of asthma treated in the method prescribed by Francis some 74 per cent. were completely relieved, 16 per cent. were greatly improved, while about 9 per cent. failed to respond at all. The method is described by Francis in the following terms:—"After painting one side of the septum nasi with a solution of cocaine and resorcin on a pledget of cotton wool attached to a probe, I draw a line with a galvano-cautery point from a spot opposite the middle turbinate body forwards and slightly downwards for a distance of rather less than half an inch. In about a week I repeat the operation on the other side, and so on at weekly intervals as occasion requires. . . . In some cases the effect can be produced by cauterising the spongy swelling which is sometimes found on each side of the posterior extremity of the septum nasi." "The results," says Mr. Robertson, "in a good many cases are dramatically prompt, and one cannot doubt the profound controlling influence of the nose in this neurosis when one sees a patient, cyanosed and almost incapable of motion, get up from the chair within five minutes quite well, possibly never to have asthma again." In illustration the author quotes the following case:—A man of about 50 years of age came to consult him in a cyanosed condition. His breathing was oppressed and wheezy, and he looked very ill. Examination of his heart and vessels pointed to the existence of renal disease, but it was decided to cauterise his septum in the hope of relieving his breathing. This was done, and the patient rose from his chair asserting that he was quite well. All dyspnœa and cyanosis had vanished. It was subsequently discovered that his urine was loaded with albumin, and that each pleural cavity contained a considerable quantity of fluid. This patient died of uræmia some months later, but the remarkable alleviation of uræmic asthma by cauterisation of the septum is justly considered by the author as a striking incident.

It is pointed out that text-books on the subject commonly insist on the necessity of correcting, in the case of asthma, any obvious deformity of the nasal passages, whether by polypi or other morbid conditions. Francis, however, insists that the best results are obtained in those cases in which the nose presents no such obvious departure from the normal, asserting further that where a small polypus is present the outlook as regards the relief of asthma is distinctly less promising whether the growth be removed or not. In the author's series there were 13 patients with polypi, of whom only five obtained complete relief.

Of other neuroses represented in the category of cases treated by the method above described hay-fever is one. The author holds that he is within the mark in asserting that at least half the cases get speedy relief, though not a few come to him once a year "to be freshened up." Heart-pain suggestive of angina appears to have yielded to the treatment in a certain number of cases. Unfortunately, the author does not give enough detail to assist the establishment of the diagnosis, but it is impossible to credit that angina vera such as is associated with organic lesions of the heart or its vessels should be, as would appear from the statement, permanently alleviated by cauterisation of the nose.

Epilepsy in association with asthma appears to have yielded in a certain number of cases, but epilepsy alone seems less responsive. But migraine responds in a fashion which the author considers to be very hopeful. "Most headaches of a periodic character," he says, "and many that are not, may be avoided by careful intra-nasal treatment. I believe that pressure of the middle turbinate on the postero-superior part of the septum is the great cause. . . . If there is no pressure, cauterise the septum; if pressure on the septum, relieve it, either by cautery or the snare. Many cases are relieved before all the pressure is removed; so there must be some spot on the septum that has control over the vaso-motor mechanism."

In discussing the *rationale* of the treatment Mr. Robertson asks what is the part played by the nose in the complicated mechanism which produces such various and divergent effects as are seen in the different neuroses considered. He assents in the view that the fundamental error is an inherent instability of the nervous system, and that, since most of these neuroses can be modified by diet alone, some metabolic toxin plays a part. He compares the role of the nose in regard to the paroxysms to that of an electric switch closing the circuit of the sympathetic vaso-motor mechanism. "I have no doubt," he observes, "that cauterisation of the septal mucosa after the method of Francis acts exactly in a similar way to a dose of nitrite of amyl or of nitroglycerine, except that its effects are more lasting."

[1] Australasian Med. Gazette, Jan. 20, 1906.

